# Psychoactive substances: screening for brief intervention in primary health care, Rio de Janeiro/Brazil

**DOI:** 10.1186/1940-0640-10-S2-P1

**Published:** 2015-09-24

**Authors:** Angela Abreu, Maria Helena Nascimento Souza, Rafael Tavares Jomar, Rafaela Costa, Pedro Parreira, Teresa Barroso

**Affiliations:** 1 Public Health Nursing, Federal University of Rio de Janeiro, Brazil; 2 State University of Rio de Janeiro, Brazil; 3 Federal University of Rio de Janeiro, Brazil; 4 Coimbra Nursing School, Portugal

## Background

About 10% of the populations in urban centers all over the world misuse psychoactive substances, independently of age, gender, education level and purchasing power. The use of these substances is direct and indirectly related with a series of health problems, among which traffic accidents, aggression, clinical depressions and conduct disorders are highlighted, besides sexual risk behavior and the risk of HIV transmission due to injectable drug use and other health problems. We analyzed the profile of a population attended in the Family Health Strategy, considering the consumption of psychoactive substances in the last three months.

## Material and methods

Quantitative, Cross-sectional descriptive survey, conducted in a community located in the north of Rio de Janeiro. The sample consisted of 1489 users of the Service, using a structured questionnaire (Alcohol, Smoking and Substance Involvement Screening Test). Data collection was conducted within one year. The analysis was performed with SPSS software using statistical measures appropriate (test t de student and ANOVA and Pearson correlations), respectively to evaluate mean differences for two and more than two groups, Pearson correlations, using the Statistical Package Social Science (SPSS) version 22.0 and we have established a level statistical significance at p <0.05. The ethical study procedures were represented by the approval of the Ethics Committee of the Municipal Secretaria of Health and Civil Defense of Rio de Janeiro number 132/09

## Results

The male population was prevalent in consumption over life and in the last three months, more frequently for tobacco use 56.4%, alcohol 75.8%, cannabis 16.9% and cocaine / crack 10.1%. Religion showed as a protective factor for drug use.

## Conclusions

We emphasize the relevance of the inclusion of the model of Brief Interventions, a low-cost and gentle technology in this area and in the nursing practice.
